# Intra- and interprofessional practices through fresh eyes: a qualitative analysis of medical students’ early workplace experiences

**DOI:** 10.1186/s12909-019-1722-8

**Published:** 2019-07-29

**Authors:** Kathleen E. Leedham-Green, Alec Knight, Rick Iedema

**Affiliations:** 1 Medical Education Research Unit, Imperial College London, Sir Alexander Fleming Building, London, SW7 2BB UK; 2 Department of Population Health Sciences, King’s College London, London, UK; 3 Centre for Team-based Practice & Learning in Health Care, King’s College London, London, UK

**Keywords:** Education, Medical, Undergraduate, Hidden curriculum, Interprofessional education, Attitudes, Socialization, Secondary care, Primary health care, Students, Medical

## Abstract

**Background:**

Professional identities are influenced by experiences in the clinical workplace including socialisation processes that may be hidden from academic faculty and potentially divergent from formal curricula. With the current educational emphasis on complexity, preparedness for practice, patient safety and team-working it is necessary to evaluate and respond to what students are learning about collaborative practices during their clinical placements.

**Methods:**

394 second year medical students at a London medical school were invited to submit a short formative essay as part of their coursework describing, evaluating and reflecting on their experiences of how healthcare professionals work together. Their experiences were derived from having spent two days each week for 25 weeks in clinical contexts across primary and secondary care. We consented 311 participants and used a Consensual Qualitative Research approach to analyse these essays, creating a ‘students-eye view’ of intra- and interprofessional practices in the workplace.

**Results:**

We identified four overarching themes in students’ essays:

- Theme 1: analyses of contextual factors driving team tensions including staff shortages, shifting teams, and infrastructural issues;
- Theme 2: observations of hierarchical and paternalistic attitudes and behaviours;
- Theme 3: respect for team members’ ability to manage and mitigate tensions and attitudes; and
- Theme 4: take-forward learning including enthusiasm for quality improvement and system change.

**Conclusions:**

Students are being socialised into a complex, hierarchical, pressurised clinical workplace and experience wide variations in professional behaviours and practices. They articulate a need to find constructive ways forward in the interests of staff wellbeing and patient care. We present educational recommendations including providing safe reflective spaces, using students’ lived experience as raw material for systems thinking and quality improvement, and closing the feedback loop with placement sites on behalf of students.

## Background

Much of a medical student’s education evolves in the clinical workplace rather than the lecture theatre, classroom or laboratory. It is in this context that their professional identity emerges [1] where they experience medicine’s tasks, vocabulary, and organising principles [2], and where professional role modelling and the construction of sociocultural norms occur [3]. This type of learning may happen out of sight of academic faculty, and it is difficult to assess its influence on student development [4]. Our study’s aim was to give voice to students’ early workplace learning about collaborative norms and practices, both within and between professions. A secondary aim was to gain insight into workplace practices and interprofessional working through a ‘fresh pair of eyes’; that is, the untutored eyes of junior medical students.

Interprofessional teamwork and collaborative practices in healthcare are taking on increased educational and practical importance. This is because of rising care complexity [5] resulting from multi-morbidity, an ageing population, workforce challenges, bureaucratic pressures, financial constraints, scientific uncertainty and technological change [6]. To navigate this complexity, healthcare professionals need to collaborate and communicate in ways that make possible the navigation and management of emergent and unpredictable circumstances [7].

When healthcare professional students are sent on clinical placements, they experience rising care complexity first hand as insider–outsiders: as people who find themselves in a liminal space as they vacillate between uninitiated–outsider and established–insider roles. As the term ‘liminal’ is intended to signal, this space is not simply a marginal, less prominent space, but can also be a privileged one. Lave and Wenger describe how inhabiting the periphery may be rewarded with a provisional legitimacy and, thanks to that, critical learning [8]. Moreover, being a novice may provide opportunities for novel perspectives on practices and processes that might otherwise remain unquestioned. Students on placements are in a unique position of being allowed into healthcare settings, to observe, and at times participate as apprentices. Learning in this context means becoming aware of and absorbing the practical dimensions of healthcare provision. While ordinarily expected to reflect on patient presentations and clinical questions, students are less commonly given the opportunity to reflect on organisational, interprofessional, cultural and relational dynamics.

There is as yet limited research on the impact of these latter workplace dynamics on medical students’ learning. Rees and colleagues [9] highlighted intergroup differences in the informal interprofessional workplace learning that occurs among students and clinicians in medicine, nursing and other healthcare disciplines. Individual and group interviews were conducted with students and clinicians across six healthcare professions, including five medical students. While this study highlighted a broad range of professional perspectives, our aim was to provide greater depth but a narrower focus: specifically, informal workplace learning from the perspective of medical students.

In another recent article, Shaw and colleagues [10] describe students’ experiences of dissonance with regard to seniors’ conducts “that contradict their [students’] ethical, moral and professional understandings of appropriate medical practice” (p. 45). Exploring the impact of this on students’ behaviour, they examined acts of covert and overt resistance on the part of students to established ways of working among seniors, concluding that it is imperative for “all workplace-learning stakeholders to better understand the social dynamics of hierarchies and resistance” (p. 45). While Shaw and colleagues focused on acts of resistance to seniors’ conducts, we aim to look more broadly at how medical students characterise the workplace sociocultural context and the interprofessional behaviours and practices encountered therein. In doing so, we acknowledge that medical students’ reflections may not constitute an ‘objective’ view: it is their perspective that is the focus of our analysis. Students may feel reluctant to be critical, and their descriptions will be influenced by memory, perspective and interpretation. This notwithstanding, their writing draws our attention to dimensions of clinical work ordinarily backgrounded in medical curricula, providing insights into the behaviours and processes that medical students find remarkable, and into how they process what they have seen.

## Methods

### Conceptual framework

Our analysis draws on Haidet and Teal’s conceptual framework for deconstructing hidden curricula in medical education [11]. This model approaches the hidden curriculum from three angles: its formation (“what are the forces fostering the hidden curriculum?”), description (“what is the hidden curriculum?”) and impact (“what are the effects of the hidden curriculum?”). For the purposes of this article, we regard the hidden curriculum to be what students learn in addition to what they are directly or deliberately taught through timetabled education. This includes socialisation, or initiation, into the workplace culture. The process of initiation may be transformational: changing the way students think and act in a fundamental way. Its description benefits from Mezirow’s [12] theory according to which transformative learning happens as students process cognitive dissonances: mismatches between what they observe, and what they feel should have happened, or their prior conceptions of what should be. Dissonances are likely to be stronger for those entering a new socio-cultural context, such as medical students entering the clinical workplace for the first time. Such dissonances tend to arise for all new entrants into a society or profession as they transition between who they were (outsiders) towards who they will or are expected to become (insiders) [13]. It is by wrestling to resolve these dissonances that students learn who they are in relation to others: a core component of identity formation. Our study sought to make explicit these ordinarily hidden dissonances and transformations.

### Study design

Students were invited to submit a formative essay assignment as part of their coursework, describing and reflecting on their experiences of how professionals work together. The assignment was not compulsory and was designed to feed forward into a small group discussion on collaborative working the following week, facilitated by their GP tutor. We analysed a random selection of essays using a Consensual Qualitative Research approach [14].

### Research team

Our research team were all trained and experienced qualitative researchers, and comprised a postdoctoral psychologist (AK) and a professor of team-based practice (RI), who were unconnected to the module, and the educational lead for the primary care module (KLG). The potential bias of (KLG) as module lead was mitigated through reflexive discussions and oversight by (AK) and (RI).

### Participants

All second year medical students in the 2017–18 academic year (*N* = 394) at King’s College London GKT School of Medical Education were eligible to participate. We did not collect demographic data, however these are likely to reflect those of the institution described elsewhere [15]. Students received their assignment in week 25 of a 36-week term having spent one day each week in primary care at one of over 40 networked general practices, and one day each week in secondary care rotating between specialities and services across four teaching hospitals (i.e. a total of 50 clinical days). Their formal interprofessional education to date included four campus-based multi-professional workshops on collaborative practices for patient safety, where they explored a range of commonly used structured practices and theoretical frameworks, assessed through team presentation.

### Data generation

As part of their coursework, students were invited to submit a formative 500–1000 word written assignment via their virtual learning environment as follows:

*“You have spent most of your time so far thinking about how healthcare professionals interact with patients. We’d like you to spend a bit of time instead thinking about how healthcare professionals interact with each other. You may have witnessed ways of working, communicating and collaborating that impress or trouble you. You may like to focus on how people communicate, or on the general processes and systems that you have observed. You may have suggestions that could improve the way healthcare professionals work together. Please describe anything you have seen and how it made you feel, thinking critically about it. You may make suggestions about how things could be done better, or about excellent practice that you feel should be replicated.”*

We invited students to share their anonymised assignments for research purposes at the point of submission, with links to the participant information sheet. 322 essays were submitted (81.7% of the year group) and 311 students consented to participate (78.9%). The high completion rate may relate to the assignment feeding forward into their weekly general practice teaching. The high consent rate may relate to our opt-out rather than opt-in consent process for analysis of routine assignments. Students were encouraged to review and update an online written consent form until the end of term. We took care to ensure that students could voice their learning without fear of repercussion. An administrator removed non-consented essays from our dataset and redacted potentially identifiable content. Ethical approval was granted by the Research Ethics Committee of King’s College London Ethics Ref: LRS-17/18–4884.

### Data analysis

#### Stage 1: data preparation and immersion

Analysis began with immersion in the data. We noted that the essays tended to include one or more narratives of an incident or observation of a process, with a section of critical reflection and take-forward learning. We selected batches of essays for analysis using a random number generator. Essays were parsed into ‘cases’ which each constituted a single incident or observation with associated reflection/analysis as some essays contained multiple observations. We then created a data extraction table that included case summaries (condensed observation and analysis) with links to the underlying essay and associated thematic coding.

#### Stage 2: content coding

Stage 2 involved coding the underlying essays for content facilitated by NVivo 12 software. The codes were created and organised using an iterative Consensual Qualitative Research process [14]. This involved two researchers (KLG and AK) independently coding a sample of 5 essays, meeting with a third auditing researcher (RI) to discuss and review their codings, resolve discrepancies, and agree a new composite coding scheme. The composite coding scheme was then re-applied to those 5 essays, and tested on a new sample of 5 randomly selected essays. This process was repeated until the coding scheme adequately covered the content of new essays (data saturation), which was achieved at 30 essays. (KLG) and (AK) tested the final coding scheme against a further randomly selected sample of 20 essays and no significant new codes arose.

#### Stage 3: overarching thematic analysis and verification

Stage 3 involved researchers deriving an overarching analysis that was grounded in the coding tree and the data-extraction table. This was achieved through an iterative process involving five meetings: continual sorting and resorting of the cases and codings, and comparing the underlying data to our emerging descriptive analysis. Finally, we re-immersed ourselves in the data and each researcher read one-third of the remaining portfolios to ensure all 311 essays had been read and no significant thematic features were missed.

## Results

**Fig. 1:**
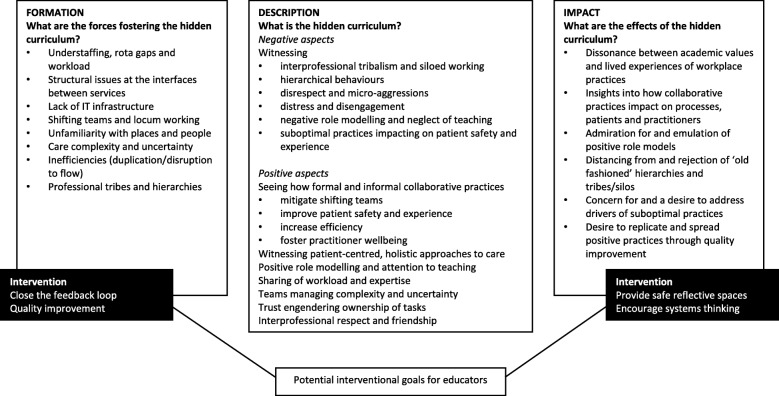
The hidden interprofessional curriculum through the eyes of medical students

We organised our data into four themes: theme 1 represents students’ sense-making of the contextual factors driving team behaviours; themes 2 and 3 evaluations of negative and positive team behaviours; and theme 4 students’ take-forward learning. These themes map onto the Formation (theme 1), Description (themes 2 and 3) and Impact (theme 4) categories of Haidet and Teal’s model for evaluating the hidden curriculum [16], and are displayed in Fig. 1.

*Theme 1: Insights into contextual factors driving team behaviours.*

Students described tensions affecting intra- and interprofessional working practices, created by shifting teams, temporary workers, rota gaps, IT infrastructure and workload. They demonstrated insights into how these impact on quality of care, patient safety and staff morale.

Students described how time pressures, overcrowding and staff shortages appeared to drive clinicians to limit their communication, rendering interprofessional boundaries more acute and problematic *“…there is often a pressure such as timing or understaffing that leads to bad communication. In these situations, individuals begin to only communicate with members of staff within their surrounding group… This leads to much confusion about the needs of individual patients and the status of each patient within the ward… Unfortunately, issues such as this seem to arise due to the pressures faced by the NHS, and not the fact that healthcare professionals are incapable of communicating with one another” (R216)*.

Understaffing was seen to affect team communication and behaviour negatively. For example, locum workers covering rota gaps were seen as creating team tensions, despite being highly qualified, as students felt they lacked knowledge about the people, structure and practices of the healthcare facility. They were seen to slow the usual flow of work as they had to keep asking others for information, in one case resulting in a patient safety incident due to unfamiliarity with local handover practices.

Students demonstrated insights into how interpersonal dynamics are shaped by organisational context, comparing fractured relationships within shifting hospital teams to more integrated and stable general practice teams: “*I have noticed that the [hospital] doctors generally get on well with each other and the nurses do also but there is not much interaction between the two groups… The team of staff working at the [GP] surgery remains much more consistent… they have the opportunity to get to know each other and become friends, not just colleagues” (R122).*

Students described how time constraints meant that staff often failed to read what others had written in the notes, affecting patient care and interprofessional dynamics. Time constraints were also seen to impact on wellbeing, when, for example, a nurse was described as not being granted time to recover from a traumatic death affecting their ability to participate fully in a meeting. Students also described how the lack of IT infrastructure, particularly at the interfaces between services, meant that work was often duplicated and expertise lost. They described teams improvising to resolve IT shortfalls, for example resolving absences and IT breakdowns using informal messaging apps.

Workforce pressures were described also as impacting on education, with some clinicians offering to teach outside their working hours, some failing to communicate absence from teaching, and others simply refusing to teach: *“…he interrupted me with” what do you want?“ quite abruptly and then when I explained that we had been asked to shadow him, he told us he didn’t have time for us…” (R078)*.

*Theme 2: Criticism of suboptimal interpersonal interactions.*

Students expressed insights into how hierarchy within professions and tribalism between professions are damaging to patient care, patient safety and staff wellbeing. For example, a junior team member was reported as unable to call on their senior for support after being abused by a patient, which was attributed to hierarchy. Consultants were observed at times to lack respect for trainee doctors: “*While all the consultants I observed were polite with patients some were less so with their juniors using them purely as typists and not engaging them at all, in the worst cases they barely even looked at them” (R027)*; and *“During a ward round I witnessed a consultant humiliate a FY1 in front of the multidisciplinary team around a patient’s bedside” (R078)*. Students were critical of teams that did not respect or support junior members of staff, or staff from other professions, with several students evaluating such practices as ‘old fashioned’.

Students reported clinicians keeping allied health professionals ‘out of the loop’, which they felt impacted on clinical care. For example, observing an interprofessional rift in a maternity unit, a student expressed concern that junior midwives appeared nervous to escalate the level of care or ask for help *(R004)*.

Students reported occasionally witnessing interprofessional and interpersonal micro-aggression, resentment and disrespect: “*I was shocked at how much of an impact issues within the healthcare team had on the communication skills of this particular doctor, but one thing for sure was that it highlighted how vital good team dynamics are for anyone working within healthcare*” *(R021)*.

Students reflected on the clash between what is taught and what is practised, noting that faculty teaching espouses respect, empathy and good communication, however in practice they experienced “*miscommunication or no communication between certain disciplines within the hospital*” *(R254)*, and practices such as “*the nurse and doctor… blaming each other for the error in front of the patient - this is not uncommon*” *(R104)*. Students also reported adversarial encounters between nursing and medical students during campus-based interprofessional education. They commented on how tribalism between the professions extended into social relationships, with friendships tending to form within but not between professions.

*Theme 3: Respect for team members’ attitudes and behaviours.*

Criticisms were greatly outnumbered by appreciative evaluations where students expressed admiration for team members who understood each other’s roles and were comfortable drawing on the expertise around them. Students described team members as mutually respectful, supporting each other, keeping the interests of the patient as their primary concern, fluidly adapting to their environment, collectively managing complexity, and with seniors supporting juniors: “*This experience made me very happy as it shows despite understaffing and the obvious strains on the NHS and healthcare professionals, with efficient management patient care can be maximised and the healthcare professionals can enjoy working in the department. It was also nice that the staff were very keen on getting myself and my fellow medical student involved and part of the team on the ward rather than an inconvenience. This experience will always stick with me in the future showing me how effective communication and collaboration can be in improving not only patient outcomes but improving the working environment of healthcare professionals*” *(R266)*.

Students described insights into how trust and ownership of a task engender responsibility and engagement, and how trust facilitates easy checking with colleagues, enhancing patient safety and effective sharing of the workload across the team. They described patient care as a uniting factor *“all the people involved in the care of a patient, from the porters to the most senior of consultants ultimately have the same goal of improving the patient’s health and experience in our healthcare system” (R083)*. There were insights into how improved cross-disciplinary communication supported practitioner wellbeing as well as patient safety and experience. For example, effective integration and cross-checking between obstetricians and midwives was seen ‘*to alleviate the tension caused in uncertain births*’ *(R004)*.

Students described and analysed many of the structured interprofessional practices that they observed such as ward rounds, board rounds, hand overs, and multidisciplinary team (MDT) meetings, praising their interprofessional participatory nature and positive impact on patient care. There were insights into how informal social communication and personal friendship mitigated against the adverse impacts of hierarchy or tribalism. Mutual respect was described as facilitating shared leadership and active participation. Students commented positively on communication taking place in different registers: informally over refreshment breaks, and formally during case reviews “*I have seen fascinating discussions happen both in meetings and other informal settings that have prevented a lot of problems that could otherwise have had a negative impact on the patient’s experience*” *(R083)*.

*Theme 4: Students’ take-forward learning.*

Students described wanting to emulate and spread interprofessional behaviours and practices that they had positively evaluated and to address the drivers of interprofessional tensions. Examples included wanting to express gratitude and praise more often, and to cross-check actions with allied professionals as well as seniors and peers. Students suggested new practices, improved practices, and the further sharing of successful practices that they witnessed elsewhere (summarised in Table 1). These new or improved approaches were suggested in response to the perceived complexities of care and the many factors detracting from practitioners being or becoming familiar with colleagues. Improved communication between and within professions was linked to patient dignity and better patient care, giving *‘patients a voice by improving communication between ourselves as health care practitioners’ (R057).*

**Table 1:** Students’ suggestions for improved interprofessional working

Relationship building	• Healthcare professionals taking time to introduce themselves to colleagues when visiting wards would help to avoid misunderstandings • Weekly multi-disciplinary team lunch could facilitate relationship building, practitioner wellbeing, and mitigate shifting teams • Active support for good relationships between different members of the MDT would help facilitate a multidisciplinary approach to problems
Patient dignity	• Interprofessional and interpersonal issues should be dealt with away from patients • Holding MDT meetings behind doors and not in the open ward would help to preserve patient confidentiality and dignity
Support for novices and juniors	• Actively inviting junior nurses, doctors and midwives to speak up and ask for help could help to overcome hierarchy • Written guides would help to support new doctors and locums to work more effectively in unfamiliar contexts • Keeping a list of patients consented for medical students to see on a ward would mean that students would not have to keep asking senior nursing staff
Interfaces between services	• Give paramedics direct access to patient records to support diagnosis, improve care and reduce admissions • Give hospitals access to primary care patient records to improve A&E efficiency and safety • Primary care doctors would benefit from direct telephone access to specialists, to consult on the effective care of complex conditions, improving patient confidence in their care • Centralising patient records would avoid duplication of scans, tests and investigations, and facilitate sharing of expertise
Collaborative practices	• Doctors and nurses could come together to explain results to patients, draw on each other’s skills, to ensure is information is both correct and communicated well • Nursing staff could proactively share the workload of surgeons during surgical admission, giving surgeons more time to respond to patient questions and concerns during the consent process • Include surgeons in MDT meetings to give them opportunities to meet new staff and to discuss the care of their patients • Enable pharmacists to join the main ward round rather than conduct their own, to help to avoid fragmentation of care and disruption to patients • Involve junior staff (as witnessed on a labour ward) to give more patients a voice through their closest carer • Institute multidisciplinary ward rounds to give junior midwives more of a voice • Multidisciplinary primary care meetings, to discuss regular or complex patients, would help to share expertise and understanding • A mid-day huddle would help ensure that developments and issues were communicated verbally across the team, reducing errors • Team huddles, rather than relying on others to find and read notes, would allow questions, improve care and facilitate the discharge process • Greater use of traffic light systems on ward whiteboards would help to communicate task prioritisation and coordinate patient discharge • ‘Parking’ questions until a handover presentation had been completed would help to ensure that there was an organised flow of information

Students described affective responses to suboptimal behaviours: “*nurses have told me that it is often the junior doctors, which don’t treat them with respect… I felt sad for the nurses when they were spoken to rudely.*” *(R219)* They expressed a desire to engage in positive change even after negative experiences: “*… after much deliberation I have come to the conclusion that these experiences have only served to provide me a deeper insight into the type of doctor I do not want to be,*” *(R254)* demonstrating engagement in critical analysis and constructive suggestions, rather than detached resignation. Students’ affective response to positively evaluated team behaviours was linked to an alignment towards and identification with those behaviours: *“The brilliant communication on the labour ward made me feel extremely proud that the concepts of patient centeredness and team work are thriving within clinical practice, and that as a profession, we have distanced ourselves from the previous style of paternalistic medicine that too often affected patient interactions.” (R057).*

Students described role modelling, interprofessional education, multi-professional socialisation and previous work experience in ancillary roles as positively influencing their attitudes to and relationships with allied healthcare professionals. As they had rotated through multiple sites and services, students were able to compare different interprofessional practices and to identify outlying examples for good or suboptimal practice. Having witnessed ‘best practice’ or a ‘good idea’, students were keen to recommend this for adoption across the system. Suggestions included team lunches and other social opportunities to build functioning teams, proactive engagement of juniors by team leaders, verbal team updates/huddles, effective use of ward whiteboards to improve task prioritisation and flow, and ward rounds and meetings that actively include the wider multidisciplinary team.

Students discussed how the benefits of multidisciplinary working also enhance primary care, suggesting and witnessing MDT approaches to complex patients. They advocated shared electronic records between primary, secondary and emergency services; and dedicated communication channels to facilitate interdisciplinary discussions between GPs and specialists “*to reach an appropriate specialist within minutes instead of having to write to the consultant and waiting days for a response or sending the patient to hospital*” *(R166)*. Some students not only proposed improvement ideas but also researched evidence and constructed detailed plans for implementation.

## Discussion

This article has set out the overarching findings derived from a study of second-year medical students’ responses to a formative task to describe, evaluate and reflect on experiences of intra- and interprofessional working. Students’ essays highlight the culture into which they are being socialised: one of contextual tensions driven by workforce pressures and infrastructural challenges, as well as hierarchical and tribal social structures. Their essays reveal some of the processes by which students make sense of their experiences of interprofessional practices in the workplace. We have shown that, faced with mixed messages, students are able to question and criticise practices where they deem them to be ineffective or suboptimal, and that they will identify with and aspire to emulate examples of good practice and positive relationships. Students link mutually respectful and trusting collaborative practices and processes to safer and more efficient patient care and to better staff engagement and morale. Their analysis of contextual tensions translates into qualified criticism of some aspects of care, but also enthusiasm for quality improvement and system change.

The research literature offers as yet limited studies of students’ views on the quality of interprofessional collaboration and communication experienced during their placements. Structured summative reflection tends to be limited in scope and uncritical of the interactive and communicative behaviours observed by students [17] [18] with criticality potentially compromised by writing for a desired grade [19]. Our research supports the few studies that have invited students to comment on the interprofessional dynamics encountered in the workplace [20] [21] which consistently highlight the importance of clinicians’ ability to enact positive and productive relationships, and to engage in effective teamwork. Our findings suggest that medical students view inclusivity within and between professions as a key component of safe, effective clinical practice, echoed in a recent study on expertise in anaesthetics [22].

Our research further highlights the engagement of these junior students in voicing their criticisms, praising and adopting the practices that support good patient care, and providing both creative and constructive suggestions for improvement. Interestingly, although students were able to analyse and make suggestions for change, none described engaging team members in conversations about change. Contrary to Shaw’s findings [10] we encountered neither reports of nor desires for acts of resistance. This may be due to the junior status of these second year medical students. Our study clarified that peripheral participation granted these students a fresh perspective on old problems, but also that it offered them limited legitimacy and few opportunities to suggest, let alone initiate, change. Medical students’ liminality requires constant monitoring and their legitimacy nurturing.

While resistance to unacceptable conduct by seniors no doubt plays a role in safeguarding novices’ moral–ethical integrity, the present study suggests we may need to support students in developing an alternative response to witnessing suboptimal behaviours. Students showed themselves capable of analysing ‘why good people do bad things’ and adopting a systems approach to explain contextual drivers to social tensions. Team behaviours do not result solely from an individual’s decisions and attitudes, but are also rooted in more broad-ranging and pervasive organisational culture, team dynamics and contexts [23]. These students were also able to compare suboptimal experiences to more positive ones, analysing how experts successfully navigate challenging circumstances through team approaches.

On this basis, we argue that placing an expectation on students to ‘resist’ may prove unworkable and unnecessary in practice. It is perhaps not practical given their position in a complex, interconnected and hierarchical workplace. If students feel unable to resist, there is the potential for disengagement, disillusionment and ‘moral erosion,’ particularly if they are unable to reconcile the cognitive dissonance between the ideal and what they see practised.

Our essay task created a safe space for students to articulate contextual, social and procedural drivers and to consider how experts mitigate these tensions in the interests of patient care. This, we suggest, may have enabled negotiation of more constructive ways forward to witnessing suboptimal practices than withdrawal or resistance. Our findings suggest that students share an interest in practice improvement and team strengthening, and that they are able to learn constructively from both positive and negative experiences: a finding that is both heartening and significant.

We do not claim that students’ essays are an accurate representation of professional behaviours or that students’ suggestions for improvement are realistic or actionable. Students were not trained ethnographers, and may lack adequate self-awareness or contextual insights. Furthermore, they will have complex relationships with their placement sites and clinical teachers: they may have felt anxious to report shortcomings, or that reflecting honestly could be held against them. Reticence to reflect openly may have been heightened as the assignment was set during the high-profile court case of Dr. Bawa-Garba [24, 25]. The assignment was designed to feed into a small group discussion with their GP tutor the following week, and their reflections will have been written for this audience.

The task engaged over 300 students in recording observations of teamwork during their placements, which may have achieved several critical aims. First, enabling students to articulate their experiences of care as a social process, focusing not on clinical-technical detail but on context, relationships, feelings and practices. Second, constructively engaging students in an analysis of team behaviours ‘as practised’ rather than ‘as ideal’ added authenticity to the learning activity. Third, students who completed the assignment would be primed to share, and thereby consolidate their sensitivities and strategies regarding team behaviours at the follow-up workshop. This could have potentially enhanced the depth of discussion there, however these workshops were not included in our research. Finally, the activity takes steps to address and evidence some of the more complex educational outcomes required by the regulators of doctors in the UK [26]. These outcomes emphasise collaborative practices under conditions of complexity and diversity, communication about matters including clinical-technical as well as socio-cultural and moral-ethical dilemmas, and the ability to negotiate ways forward amidst inevitable uncertainty and unavoidable compromise.

## Conclusions

We hope that this study will support healthcare practitioners in reflecting on interprofessional culture and practices, and the factors that drive team behaviours in the clinical environment. Educators, however, may ask how they can enable students to respond positively to what they see, and to develop the skills to navigate an uncertain, changing and occasionally dysfunctional workplace. Rather than teaching an idealised form of communication driven by academic values, we suggest that educators start by asking students to study and respond to the realities of the workplace context and culture.

The assignment itself appears to have contributed meaningfully to students’ learning about workplace cultures and relationships: two facets of healthcare education that are considered key in the care of patients, yet difficult to define, teach or assess, other than through abstract principles and distant ideals [27]. Educators might consider providing similar safe, reflective spaces for students to articulate and process their experiences of intra- and interprofessional working, beginning with students’ lived experiences of workplace practices as raw material for systems thinking and service analysis.

A final legitimisation of seeking students’ views would be to integrate them into feedback to students’ placement sites as potential topics for quality improvement and change. This would obviate the unrealistic scenario where novices are expected to challenge established practices and professional hierarchies. It would however require medical schools to adopt a culture that is responsive and open towards their students, and to act as their advocates in a learning process with placement providers.

## Data Availability

The datasets generated and/or analysed during the current study are not publicly available due to concern that it would enable establishments or individuals to be identified due to rich contextual detail. Anonymised redacted transcripts are available from the corresponding author on reasonable request on condition that they are not published in their entirety. Please direct requests to the corresponding author.

## Abbreviations

GP: General Practitioner
IT: Information Technology
MDT: Multi-Disciplinary Team
